# Emotional intelligence matters in hospitality education: contributions of emotional intelligence, fluid ability, and personality to hospitality grades

**DOI:** 10.3389/fpsyg.2023.1148863

**Published:** 2023-04-26

**Authors:** Juliane Völker, Inès Blal, Marcello Mortillaro

**Affiliations:** ^1^Swiss Center for Affective Sciences, University of Geneva, Geneva, Switzerland; ^2^EHL Hospitality Business School, HES-SO//Haute École Specialisée de Suisse Occidentale, Lausanne, Switzerland

**Keywords:** ability emotional intelligence, academic performance, fluid ability, hospitality education, personality

## Abstract

**Introduction:**

According to recent meta-analyses, emotional intelligence can significantly predict academic performance. In this research, we wanted to investigate a particular group of students for which emotional intelligence should be crucial. Namely, we examined whether emotional intelligence, conceptualized as an ability, uniquely contributes to academic performance in hospitality management education beyond fluid intelligence and personality.

**Methods:**

Using a battery of tests and questionnaires in an online survey, we analyzed if fluid ability, the Big-Five personality dimensions, and ability-based emotional intelligence predict six module grades in a sample of N = 330 first-semester students at a Swiss-based hospitality school.

**Results:**

We found that the ability to manage other people’s emotions is more predictive of module grades than fluid ability if the courses involve substantial parts of interactive work. Complementarily, the more a module focuses on theoretical knowledge or abstract subject material, the more fluid ability predicted performance. Other abilities and factors – emotion understanding, emotion regulation, the students’ age, conscientiousness, and openness – predicted performance only in specific modules, hinting that the didactic methods and grading procedures are complex and involve various skills and dispositions of the students.

**Discussion:**

Given that the hospitality education and industry are buzzing with interactions with peers and guests alike, we provide evidence that interpersonal and emotional competencies are vital to hospitality curricula.

## Introduction

Research in the field of higher education is replete with studies that point to cognitive intelligence as the single most important factor in predicting an individual’s academic performance, most frequently measured by GPA score or SAT results (Mohzan et al., 2013; Ahmed et al., 2019). Notwithstanding its long-lasting predominance in the academic education literature and its indisputable relevance, this traditional stream of research is regularly being adjourned by a growing body of studies focusing on measuring the impact of other skills on academic performance in higher education settings (e.g., Rozell et al., 2002; Jaeger and Eagan, 2007; Nasir and Masrur, 2010; Shipley et al., 2010; Brackett et al., 2011; Sánchez et al., 2013; Wang, 2019; MacCann et al., 2020; Zhoc et al., 2020; Goh and Kim, 2021). Persistence, motivation, determination, stress management, and engaging in fruitful relationships with academic instructors and classmates are all associated with academic performance but also relate to the construct of emotional intelligence (Goh and Kim, 2021).

The term ‘emotional intelligence’ (EI) was coined by Salovey and Mayer (1990), who defined the concept as the ability to perceive, understand, regulate, and harness emotions in the self and others. From this original definition emerged studies that showed that EI could be measured as an ability (Ciarrochi et al., 2000; Mayer et al., 2016) or as a personality trait (Petrides et al., 2007). Extant research provides evidence that just like cognitive intelligence and personality, EI is positively associated with academic performance, job behavior, and success in life (van Rooy and Viswesvaran, 2004; Richardson et al., 2012; Perera and DiGiacomo, 2013; Sánchez et al., 2013; Wolfe and Kim, 2013; Choi et al., 2019; Zhoc et al., 2020). Educational environments require students to acquire and employ knowledge, study diligently, develop competencies, and be curious and critical. In these environments, students engage with their study material independently, but they must also interact with peers and teachers to attain good grades. Past research has established two groups of predictors of academic achievement: cognitive intelligence and personality traits. Cognitive intelligence explains a student’s capacity to reason about (novel) material, grasp abstract concepts, and use them to build knowledge. As such, intelligence is a stable predictor of grades (Laidra et al., 2007; Bergold and Steinmayr, 2018). However, a large capacity for learning is not always sufficient for achievement, and students’ personality traits come into play, influencing their approaches toward learning. Students’ conscientiousness (their willingness to work hard) was observed to be a prime predictor of academic achievement alongside cognitive intelligence, yet important roles are also found in openness (their curiosity and motivation to engage with subject material) and agreeableness (their tendency to behave in cooperative ways; Duff et al., 2004; Poropat, 2009; Verbree et al., 2021). With capacity (intelligence) and willingness (personality) providing essential contributors to academic grades, the picture of academic success seemed still incomplete, especially if learning environments are emotional, focus on developing individual competencies and interpersonal skills, and bustle with human interaction. Therefore, the potential contributions of EI to academic achievement have garnered more and more interest (Wolfe and Kim, 2013; Wolfe et al., 2014; Wilson-Wünsch et al., 2016).

MacCann et al. (2020) recently published a comprehensive large-scale meta-analysis (anchored in both high-school and university contexts) that estimates the extent to which EI predicts academic performance. Their analysis revealed a small to moderate association between EI and academic performance. The strength of this association was related to how EI was conceptualized and measured: EI was a stronger predictor of academic performance when measured using the ability approach (i.e., skill-based), as opposed to the ‘self-rated’ or ‘mixed’ approaches that lean closer toward personality-based concepts.

MacCann et al. (2020) suggest that the link between EI and academic achievement is threefold: First, EI helps cope with emotions in academic life (e.g., manage stress and stay motivated). Coherently with this interpretation, Mohzan et al. (2013) showed that students who are more confident that they understand and appraise their own emotions correctly also have a better propensity to regulate themselves in a stressful education environment. Zhoc et al. (2020) discovered that a higher self-reported EI translates to better academic performance *via* stronger cognitive and social engagement at university, which enables students to make university life experiences and challenges positive and rewarding. What is more, hospitality students who scored higher in self-reported EI seem more likely to pursue a career in hospitality (Walsh et al., 2015).

Second, EI is beneficial for building relationships while at university. For example, Goh and Kim (2021) reported that the academic performance of hotel management students was associated with emotionality, a facet of trait EI. They argue that emotionality (i.e., the subjective capacity to perceive and express emotions adequately) helps students to work effectively with others on group assignments and build a supportive network with classmates and teachers.

Third, EI is related to academic achievement because when measured as an ability, it moderately overlaps with cognitive intelligence (MacCann et al., 2014), thereby forming an association with grades. In a meta-analysis, Miao et al. (2019) report strong associations between service quality and all models of EI, yet the association is most substantial with ability EI.

Because EI seems to cover what intelligence and personality do not (sufficiently), hospitality professionals have demonstrated interest in integrating emotional competencies into hospitality curricula. Promoting EI should propagate the students’ professional performance as they are more adept at dealing with emotions on the job (their own, their peers’, and customers’), and it also brings benefits to the classroom: In the learning setting, the basis for developing skills and competencies is mutual support, a good learning atmosphere, and a rewarding social life (Gibbs and Slevitch, 2019;Wolfe et al., 2014).

Taken together, we can confidently conclude that students’ cognitive ability and personality affect their academic performance. EI also contributes to academic achievement, but if measured *via* self-report, it taps more into personality and less into the ability space. Some researchers raised concerns about the suitability of self-report measures to describe actual emotional competencies in the hospitality domain (Boz and Koc, 2021). However, we are unaware of studies in hospitality management education that employed an ability measure of EI. Thus, we aimed to elucidate the association between a performance-based measure of ability EI and the academic performance of hospitality students. We investigated the predictive power of single emotional abilities (as opposed to a global EI score) beyond cognitive ability and personality regarding several module grades of hospitality students using hierarchical regression analyses. We expect EI to explain distinct portions of variance in the grades beyond cognitive ability and personality.

The results of our study can have many relevant implications for researchers and practitioners. In theoretical considerations, we can further develop our understanding of how ability EI complements cognitive intelligence and personality in predicting academic success. Namely, we aim to elucidate the critical emotional component embedded in almost any human interaction in the context of hospitality. In practical terms, the focus on specific EI competencies - assessed with performance-based measures - contributes valuable indications for hospitality professionals (Wolfe et al., 2014; Koc and Boz, 2020). Thus, it is useful to see whether and which competencies identified by ability EI can provide unique advantages and therefore be used to inform hospitality education practices and curricula (Wolfe, 2017).

## Method

We examined how EI (i.e., emotion recognition, understanding, regulation, and management), fluid ability, and personality (extraversion, agreeableness, conscientiousness, emotional stability, and openness) predict the grades of hospitality school students in six different modules.

### Participants and procedure

The participants were first-semester students at the École Hôtelière de Lausanne (EHL), an internationally renowned hospitality school in Switzerland. Students were recruited at the start of each academic year between fall 2019 and fall 2021 (five cohorts: students can start with the fall or spring semester). The students completed two online surveys administered *via* Qualtrics.^1^ The first survey assessed emotional abilities, and the second survey fluid ability and personality. Students did not receive monetary compensation for their participation, but each cohort had a debriefing workshop during their last semester at the school. By the end of the first semester, the hospitality school provided the anonymized students’ grades. In total, 642 students completed the emotional abilities survey, and 416 students finished the fluid ability and personality survey. Finally, 330 students’ data were complete. The final sample comprised 217 females and 113 males. Age ranged from 18 to 29 years, with a mean age of 19.64 years (*SD* = 1.45). Nearly half of the students originated from Switzerland (*n* = 137; 41.5%), yet most indicated nationalities outside Switzerland (*n* = 193, 58.5%). All participants provided signed consent.

### Measures

#### Module grades

The hospitality school provided the grades of six modules (Table 1). Within each module, students attended one to six courses. The mean course grade constitutes the final module grade. The courses teach various skills related to business management, administration, and technical skills in gastronomy and housekeeping. Depending on the subject matter of the module, the coursework differs in the degrees of practical and theoretical work. For example, in the modules *Food & Beverage workshops I*, *II*, and *Wine & Spirits*, activities involve team exercises, taking on shifts in local restaurants, but grading also includes teachers’ evaluation of students’ transferable skills (e.g., teamwork or attitude).

**Table 1:** Modules and related courses.

Module	Courses	Activities	Gradings
Food & Beverage workshops I	Bakery and pastry making Catering Events: management and operations Fine dining cuisine Fine dining restaurant and lounge bar	90% practical (group exercises), 10% theory	MCQ, transferable skills^1^
Food & Beverage workshops II	Bar and restaurant outlets International cuisine R&D design lab Stewarding	90% practical (group exercises, shift work), 10% theory	MCQ, transferable skills^1^
Wine & spirits	Global spirits Oenology and wine-producing regions	90% practical (workshops, tastings), 10% theory	Written exams
Rooms division	Front office and spa Housekeeping	50% practical (operational tasks, service management), 50% theory	MCQ, transferable skills^1^
Introduction to business tools	Applied mathematics and Excel for business hospitality management	100% theory (autonomous study, online lessons)	PC-based assessment, quiz
Introduction to hospitality management	French^2^ Introduction to Business English^2^ Hospitality concept discovery Introduction to F&B management Rooms division operations Sustainable hospitality culture	100% theory (lectures, work in class, excursion)	Written exams

MCQ = multiple choice questionnaire.

1 Teachers evaluate the students’ attitude, teamwork and communication, self-development, and other course-specific criteria (e.g., development of versatility) by awarding points.

2 Language courses were mandatory only for specific students (e.g., native French speakers did not have to take French); therefore, not all students received grades on these courses.

In contrast, the modules *Introduction to business tools* and *Introduction to hospitality management* comprise mostly business school subject material that students acquire by attending classes, lectures, or engaging in self-study. The grading system ranges from 1 (lowest score) to 6 (highest score), with scores below 4 indicating a failed course. If students did not complete a module and received no grade, we coded their grade as a missing value.

#### Emotional abilities

Students completed the Geneva emotional competence test (GECo; Schlegel and Mortillaro, 2019) in English (*n* = 285) or French (*n* = 43). The GECo assesses four emotional abilities. First, in the emotion recognition subtest, short video clips are presented in which actors use para-and non-verbal behavior to portray a specific emotion. Participants must choose the correct answer from a list of 14 emotions (see also GERT-S; Schlegel and Scherer, 2016). Second, the subtest on emotion understanding presents short vignettes that describe various contexts, sometimes including components of emotions (e.g., descriptions of physiological phenomena or cognitive appraisals of the situation). Using the information presented in the vignette, the participants must deduce the correct emotion felt by the protagonist. The correct answers are based on the component-process model of emotion (Scherer et al., 2001). Third, in the emotion regulation subtest, short scenarios are presented, and the participant is asked to imagine him-or herself in that situation and indicate two behaviors that they would most likely show to minimize negative emotions. In each scenario, the responses represent two adaptive and two maladaptive regulation strategies, according to Garnefski et al. (2001). Finally, the emotion management subtest presents workplace conflict scenarios in which another person experiences anger, fear, sadness, or inappropriate joy. The participant is asked to select one response they would typically show to influence the emotion of the other person. The responses reflect the conflict management strategies described by Thomas (1992). The correct option is the one that is appropriate in consideration of various situational factors, such as time pressure, stakes, or organizational norms. The GECo takes about 50 to 60 min to complete and provides scores for each subtest and a total score from 0 to 1.

#### Fluid ability

Psychometric and neuroimaging evidence suggests a vast overlap of fluid ability and general intelligence, making it a reliable estimate of overall cognitive ability (Blair, 2006; Bergold and Steinmayr, 2018). We employed the English version of the Culture Fair Intelligence Test (CFT; Cattell and Cattell, 1957) to measure fluid ability. The CFT assesses the ability to analyze figure series, classify figures, solve figure matrices, and infer rules from figural presentations. Participants were presented with the four series of tasks and asked to solve as many as possible within 3 min for each subtest (4 min on the fourth subtest). The CFT comprises 56 tasks, and scores can range from 0 to 56. The total time to complete the CFT is about 15 min.

#### Personality

We used the English (*n* = 282) or French version (*n* = 48) of the Ten Item Personality Inventory (TIPI; Gosling et al., 2003) to assess extraversion, agreeableness, conscientiousness, emotional stability (reverse neuroticism), and openness. Participants indicated their agreement to sentences describing themselves with different adjectives on a scale from 1 (*completely disagree*) to 7 (*completely agree*). We report mean scores for each personality dimension. The TIPI takes about 5 min to complete.

## Results

All data was used. Missing values (i.e., incomplete module grades) were excluded pairwise (correlations) or listwise (regression). Analyses were run in IBM SPSS version 27. First, we report descriptive statistics of the module grades and examine the influences of students’ gender, age, and nationality. Then we report descriptive statistics of fluid ability, personality dimensions, and emotional abilities, and again analyze relationships with gender, age, and nationality. Finally, we report hierarchical regression analyses of the predictors (step 1: demographics, step 2: fluid ability and personality, step 3: emotional abilities) onto the six module grades to determine any incremental validity of emotional abilities beyond fluid ability and personality.

### Module grades

Means, reliabilities, and correlations of the module grades are presented in Table 2. Some modules only incorporate a few courses, so we calculated split-half reliabilities (Spearman-Brown coefficient) to gage the consistency of the course grades used to compute each module grade. Reliabilities range from 0.481 (*Introduction to hospitality management*) to 0.675 (*Food & Beverage workshops I*), indicating that the course grades within one module have some degree of heterogeneity. In other words, if a student receives a good grade in one course, they do not necessarily receive a good grade in another course of that same module. This variability can derive from the differences between the courses within a module in terms of content, structure, delivery mode, or grading procedure. All module grades are moderately or highly correlated, ranging from *r* = 0.335 to *r* = 0.716 (all *p*s < 0.001).

**Table 2:** Module grades: descriptive statistics, internal consistencies, and correlations.

Module	*N*	*M* (*SD*)	Reliability^1^	Correlations				
				1.	2.	3.	4.	5.
1. Food & Beverage workshops I	298	4.85 (0.38)	0.675					
2. Food & Beverage workshops II	308	4.87 (0.46)	0.616	0.716***				
3. Wine & spirits	320	5.00 (0.57)	0.651	0.623***	0.637***			
4. Rooms division	323	5.18 (0.42)	0.548	0.612***	0.663***	0.618***		
5. Introduction to business tools	316	4.62 (0.98)	–	0.420***	0.335***	0.385***	0.400***	
6. Introduction to hospitality management	326	4.78 (0.47)	0.481	0.466***	0.471***	0.513***	0.520***	0.491***

*N* = 330. Grades range from 1 (low) to 6 (high). Ns vary because modules were only graded if a student completed all mandatory courses within each module.

1 Course grades (*cf.* Table 1) were used to compute Spearman-Brown Coefficients for each module. In *Introduction to hospitality management*, the grade on *French* was excluded because otherwise no valid cases were identified (no student completed all six available courses); if taken, it contributed to the module grade as per the hospitality school’s regulations. No coefficient was calculated for *Introduction to business tools* because the module comprised only one course in the first semester.

***p < 0.001.

Males achieved slightly better grades on *Introduction to business tools* (*M* = 4.77, *SE* = 0.081) than females (*M* = 4.53, *SE* = 0.072), *F*(1, 314) = 4.39, *p* = 0.037, η^2^ = 0.014. Age correlated with the grades on *Food & Beverage workshops I* (*r* = 0.199, *p* = 0.001) and *II* (*r* = 0.201, *p* < 0.001), *Rooms division* (*r* = 0.177, *p* = 0.001), and *Wine & Spirits* (*r* = 0.156, *p* = 0.005). Comparing Swiss to non-Swiss nationalities, participants from Switzerland received higher grades in three modules: First, in *Wine & Spirits* (*M* = 5.11, *SE* = 0.044; compared to *M* = 4.92, *SE* = 0.044), *F*(1, 318) = 9.15, *p* = 0.003, η^2^ = 0.028. Second, in *Food & Beverage workshops I* (*M* = 4.93, *SE* = 0.035; compared to *M* = 4.80, *SE* = 0.028), *F*(1, 296) = 7.99, *p* = 0.005, η^2^ = 0.026. Finally, also in *Food & Beverage workshops II* (*M* = 4.93, *SE* = 0.044; compared to *M* = 4.82, *SE* = 0.032), *F*(1, 306) = 4.60, *p* = 0.033, η^2^ = 0.015.

### Predictors

Means, reliabilities, and correlations of fluid ability, personality, and emotional abilities are presented in Table 3. We calculated McDonald’s ω (Hayes and Coutts, 2020) to estimate the reliabilities of the GECo and CFT. Reliabilities ranged from acceptable to good, with estimates on emotion regulation (0.485) and emotion management (0.607) evoking some caution. Because the TIPI uses only two items per dimension, we calculated Spearman-Brown Coefficients. Reliability was good for conscientiousness (0.727), extraversion (0.640), and openness (0.690), barely acceptable for emotional stability (0.501), and low for agreeableness (0.224).

**Table 3:** Fluid ability, personality, and emotional abilities: descriptive statistics, internal consistencies, and correlations.

Measure	*M* (*SD*)	Reliability^1^	Correlations								
			1.	2.	3.	4.	5.	6.	7.	8.	9.
1. Fluid abilities	35.45 (8.03)	0.847									
2. Extraversion	4.49 (1.30)	0.640	0.077								
3. Agreeableness	4.58 (1.23)	0.224	−0.030	0.251***							
4. Conscientiousness	5.05 (1.57)	0.727	−0.048	0.317***	0.485***						
5. Emotional Stability	3.96 (1.39)	0.501	−0.011	0.106	0.075	0.090					
6. Openness	5.17 (1.42)	0.690	−0.066	0.327***	0.510***	0.596***	0.089				
7. Emotion recognition	0.582 (0.152)	0.740	0.214***	0.067	0.089	0.091	−0.001	0.125*			
8. Emotion understanding	0.658 (0.173)	0.721	0.310***	0.025	0.060	0.057	−0.108	0.001	0.387***		
9. Emotion regulation	0.587 (0.097)	0.485	0.073	0.126*	0.006	0.055	−0.008	0.015	0.046	0.102	
10. Emotion management	0.447 (0.163)	0.607	0.379***	0.032	0.149**	0.077	−0.026	0.062	0.191***	0.339***	0.236***

*N* = 330.

1 Reliability measures: CFT (fluid ability) and GECo (emotional abilities): McDonald’s ω; TIPI (personality): Spearman-Brown Coefficient.

1: CFT sum score ranges from 0 to 56; 2–6: TIPI mean scores range from 1 to 7; 7– 10: GECo mean scores range from 0 to 1.

*p < 0.05, **p < 0.01, ***p < 0.001.

Emotion recognition, emotion management, and emotion understanding significantly correlated among themselves, while emotion regulation correlated only with emotion management (*r* = 0.24, *p* < 0.001). This result has been observed before. It is argued that the regulation subtest taps into an individual’s behavior (selecting their typical response) rather than their ability (knowing what the best response would be; Völker, 2020; Simonet et al., 2021). Emotion recognition, emotion understanding, and emotion management (but not emotion regulation) were also significantly associated with fluid ability (respectively, *r* = 0.21, *r* = 0.31, *r* = 0.38, all *p*s < 0.001). Additionally, a few weak relationships between emotional abilities and personality were found. A better emotion recognition ability was associated with more openness (*r* = 0.13, *p* = 0.023); better emotion regulation was associated with extraversion (*r* = 0.13, *p* = 0.022); and higher emotion management ability was moderately associated with agreeableness (*r* = 0.15, *p* = 0.007). Fluid ability did not correlate with personality. All personality dimensions were strongly interrelated except for emotional stability, which did not correlate with any other personality dimension. Therefore, our results largely reflect previous research that proposes independence between ability and personality, with emotional abilities partly overlapping with the cognitive ability space (Schlegel and Mortillaro, 2019; MacCann et al., 2020).

No correlations with age were found. Gender did not impact the scores on either fluid or emotional abilities. On extraversion, however, males scored higher (*M* = 4.71, *SE* = 0.121) than females (*M* = 4.38, *SE* = 0.088), *F*(1, 328) = 4.77, *p* = 0.030, η^2^ = 0.014; whereas on openness, females scored higher (*M* = 5.29, *SE* = 0.089) than males (*M* = 4.94, *SE* = 0.149), *F*(1, 328) = 4.66, *p* = 0.032, η^2^ = 0.014. We found differences between Swiss and non-Swiss participants: Swiss students performed better on the emotion recognition subtest (*M* = 0.618, *SE* = 0.012; compared to *M* = 0.557, *SE* = 0.011), *F*(1, 328) = 12.91, *p* < 0.001, η^2^ = 0.038. Swiss students were also less open (*M* = 4.84, *SE* = 0.136; compared to *M* = 5.41, *SE* = 0.089), *F*(1, 328) = 13.50, *p* < 0.001, η^2^ = 0.040; less agreeable (*M* = 4.39, *SE* = 0.115; compared to *M* = 4.72, *SE* = 0.082), *F*(1, 328) = 5.63, *p* = 0.018, η^2^ = 0.017; and less conscientious than international students (*M* = 4.77, *SE* = 0.149; compared to *M* = 5.25, *SE* = 0.101), *F*(1, 328) = 7.81, *p* = 0.006, η^2^ = 0.023.

### Regression analyses

We performed multiple regression analyses to test the contributions of fluid ability, personality, and emotional abilities to the six module grades. Because of the relationships between grades and age, gender, and nationalities, we included these demographic variables as covariates in the first step. The second step introduced fluid ability and personality dimensions; the final step introduced emotional abilities (Table 4).

**Table 4:** Hierarchical regression of demographics, fluid ability, personality, and emotional abilities onto module grades.

Measure	Food & Beverages I			Food & Beverages II			Wine & Spirits			Rooms division		
	*B*	*SE*	*p*	*B*	*SE*	*p*	*B*	*SE*	*p*	*B*	*SE*	*p*
**Step 1**
Constant	3.98	0.306	***	3.62	0.371	***	4.02	0.454	***	4.19	0.333	***
Age	0.044	0.016	0.006**	0.064	0.019	0.001**	0.049	0.024	0.041*	0.051	0.017	0.004**
Gender	−0.058	0.045	0.207	−0.119	0.055	0.032*	−0.109	0.067	0.103	−0.072	0.050	0.146
Nationality	0.077	0.047	0.107	0.049	0.056	0.385	0.146	0.068	0.032*	0.035	0.051	0.493
*R*^2^ (Δ*R*^2^)	0.053 (0.053)			0.057 (0.057)			0.046 (0.046)			0.039 (0.039)		
Δ*F*		5.48**		6.16***			5.08**			4.32**		
**Step 2**
Constant	3.67	0.332	***	3.25	0.403	***	3.67	0.498	***	3.75	0.355	***
Age	0.042	0.016	0.008**	0.063	0.019	0.001**	0.049	0.024	0.040*	0.049	0.017	0.004**
Gender	−0.071	0.046	0.121	−0.138	0.056	0.013*	−0.125	0.068	0.065	−0.080	0.049	0.104
Nationality	0.075	0.047	0.114	0.070	0.057	0.219	0.139	0.069	0.044*	0.042	0.050	0.404
Fluid ability	0.009	0.003	0.001**	0.005	0.003	0.094	0.011	0.004	0.007**	0.013	0.003	***
Extraversion	0.015	0.018	0.394	0.040	0.022	0.068	−0.013	0.026	0.607	−0.002	0.019	0.914
Agreeableness	0.019	0.021	0.354	−0.012	0.025	0.644	0.020	0.030	0.510	0.011	0.022	0.613
Conscientiousness	0.027	0.017	0.129	0.042	0.021	0.048*	0.036	0.025	0.162	0.042	0.018	0.025*
Emotional Stability	−0.008	0.015	0.587	−0.012	0.019	0.527	0.010	0.023	0.653	−0.016	0.016	0.317
Openness	−0.043	0.020	0.038*	−0.018	0.025	0.464	−0.051	0.029	0.086	−0.036	0.021	0.091
*R*^2^ (Δ*R*^2^)	0.110 (0.057)			0.097 (0.040)			0.081 (0.035)			0.122 (0.083)		
Δ*F*		3.05**		2.20*			1.95			4.95***		
**Step 3**
Constant	3.74	0.349	***	3.32	0.421	***	3.90	0.516	***	3.79	0.371	***
Age	0.039	0.016	0.012*	0.061	0.019	0.002**	0.045	0.023	0.054	0.047	0.017	0.005**
Gender	−0.047	0.046	0.309	−0.108	0.056	0.052	−0.085	0.067	0.207	−0.069	0.049	0.159
Nationality	0.057	0.048	0.234	0.045	0.057	0.435	0.113	0.070	0.106	0.041	0.051	0.426
Fluid ability	0.005	0.003	0.090	0.000	0.004	0.957	0.004	0.004	0.403	0.009	0.003	0.003**
Extraversion	0.019	0.018	0.303	0.043	0.022	0.050	−0.006	0.026	0.820	0.002	0.019	0.907
Agreeableness	0.010	0.021	0.648	−0.026	0.025	0.291	0.002	0.030	0.940	0.001	0.022	0.952
Conscientiousness	0.025	0.017	0.151	0.040	0.021	0.058	0.032	0.025	0.196	0.039	0.018	0.036*
Emotional Stability	−0.008	0.015	0.584	−0.010	0.019	0.580	0.012	0.022	0.579	−0.011	0.016	0.481
Openness	−0.046	0.020	0.024*	−0.020	0.025	0.422	−0.054	0.029	0.065	−0.034	0.021	0.111
Emotion recognition	0.300	0.153	0.051	0.319	0.186	0.088	0.279	0.227	0.218	−0.064	0.164	0.697
Emotion understanding	−0.043	0.139	0.760	0.067	0.168	0.690	0.171	0.206	0.407	0.306	0.147	0.039*
Emotion regulation	−0.243	0.226	0.283	−0.355	0.267	0.185	−0.700	0.321	0.030*	−0.267	0.236	0.258
Emotion management	0.385	0.150	0.011*	0.481	0.181	0.008**	0.636	0.218	0.004**	0.317	0.157	0.045*
*R*^2^ (Δ*R*^2^)	0.144 (0.035)			0.135 (0.038)			0.127 (0.046)			0.153 (0.039)		
Δ*F*	2.90*			3.24*			4.03**			2.80*		

Measure	Business tools			Hospitality management		
	*B*	*SE*	*p*	*B*	*SE*	*p*
**Step 1**
Constant	4.31	0.793	***	4.21	0.376	***
Age	0.012	0.041	0.771	0.028	0.020	0.154
Gender	0.237	0.117	0.044*	−0.003	0.055	0.950
Nationality	−0.022	0.120	0.853	0.036	0.057	0.520
*R*^2^ (Δ*R*^2^)	0.014 (0.014)			0.011 (0.011)		
Δ*F*	1.48			1.23		
**Step 2**
Constant	3.23	0.824	***	3.73	0.415	***
Age	−0.005	0.039	0.909	0.027	0.020	0.162
Gender	0.178	0.113	0.116	−0.013	0.056	0.820
Nationality	−0.021	0.116	0.858	0.056	0.057	0.332
Fluid ability	0.040	0.007	***	0.007	0.003	0.034*
Extraversion	0.045	0.044	0.310	0.023	0.022	0.283
Agreeableness	0.054	0.053	0.308	0.015	0.025	0.559
Conscientiousness	0.031	0.044	0.470	0.027	0.021	0.200
Emotional Stability	−0.003	0.038	0.929	0.001	0.019	0.955
Openness	−0.113	0.049	0.023*	−0.012	0.025	0.628
*R*^2^ (Δ*R*^2^)	0.138 (0.124)			0.043 (0.031)		
Δ*F*	7.31***			1.72		
**Step 3**
Constant	3.97	0.861	***	3.72	0.439	***
Age	0.001	0.039	0.987	0.025	0.020	0.206
Gender	0.177	0.113	0.118	−0.004	0.057	0.943
Nationality	0.033	0.117	0.776	0.052	0.059	0.375
Fluid ability	0.048	0.008	***	0.004	0.004	0.222
Extraversion	0.053	0.044	0.232	0.024	0.022	0.269
Agreeableness	0.059	0.053	0.262	0.009	0.026	0.718
Conscientiousness	0.044	0.043	0.307	0.025	0.021	0.239
Emotional Stability	−0.014	0.038	0.716	0.002	0.019	0.902
Openness	−0.109	0.049	0.028*	−0.012	0.025	0.633
Emotion recognition	−0.698	0.387	0.072	0.038	0.192	0.844
Emotion understanding	−0.627	0.344	0.069	0.045	0.172	0.793
Emotion regulation	−0.753	0.545	0.168	−0.002	0.274	0.993
Emotion management	0.008	0.373	0.984	0.245	0.187	0.190
*R*^2^ (Δ*R*^2^)	0.171 (0.033)			0.050 (0.007)		
Δ*F*	3.03*			0.577		

*N* = 330.

Regression method: Enter. Step 1: Age, Gender (0 female, 1 male), Nationality (0 international, 1 Swiss); Step 2: Fluid ability, personality; Step 3: Emotional abilities.

*p < 0.05, **p < 0.01, ***p < 0.001.

Step 3 shows that the predictors explained significant portions of variance in five of the six module grades, ranging from 12.7% (*Wine & Spirits*) to 17.1% (*Introduction to business tools*). Specifically, the ability to manage emotions contributed significantly to four grades (*Wine & Spirits*, *Food & Beverage workshops I* and *II*, and *Rooms division*). Emotion regulation explained variance in *Wine & Spirits*, whereas Emotion understanding explained variance in *Rooms division*. Age explained significant portions of variance in three grades (*Food & Beverage workshops I* and *II*, *Rooms division*). Fluid ability contributed significantly to two grades (*Introduction to business tools*, *Rooms division*). Finally, openness predicted two grades (*Food & Beverage workshops I*, *Introduction to business tools*), and conscientiousness one grade (*Rooms division*). Overall, emotional abilities were significant predictors to four grades, age and personality each to three grades, and fluid ability to two grades.

To test whether emotional abilities would explain more variance in modules that involve practical rather than theoretical course activities, we computed the average grade of majorly theoretical modules (*Introduction to business tools*, *Introduction to hospitality management*; Spearman-Brown coefficient = 0.658) and practical courses (*Food & Beverage workshops I* and *II*, *Wine & Spirits*, *Rooms division*; Spearman-Brown coefficient = 0.882). The regression model remained the same as employed for the single module grades.

The predictors explained about 11% of variance in the theoretical courses, *R*^2^ = 0.109, *F*(13, 313) = 2.96, *p* < 0.001. Only fluid ability made a significant contribution (*B* = 0.022, *SE* = 0.005, *p* < 0.001). In contrast, the same model explained 17% variance in the practical courses, *R*^2^ = 0.174, *F*(13, 315) = 5.10, *p* < 0.001. The significant predictors were age (*B* = 0.051, *SE* = 0.016, *p* = 0.001), emotion management (*B* = 0.470, *SE* = 0.147, *p* = 0.002) and, approaching significance, conscientiousness (*B* = 0.034, *SE* = 0.017, *p* = 0.050). Therefore, fluid ability significantly predicted module grades that predominately involved theoretical coursework, yet in modules that involved practical coursework, the most important predictors were age and emotion management.

## Discussion

We aimed to examine the contributions of emotional abilities beyond fluid reasoning and personality to the academic performance of hospitality students during their first semester of the bachelor program. Using regression analyses, we found that emotional abilities – in particular, emotion management – contributed to the grades of modules that predominately involved practical activities. In contrast, fluid ability contributed to modules teaching theoretical subject material through less interactive didactic methods. Directly comparing average grades of theoretical to practical modules, the difference in the variance explained was remarkable (11% vs. 17%). On top of that, we observed small contributions of personality, similar to the results of Wilson-Wünsch et al. (2016) and Verbree et al. (2021): lower openness and higher conscientiousness benefitted specific grades. We discuss and interpret the findings below.

### Practical modules

The ability model of EI covers different emotional abilities (Mayer et al., 2016). We found that these abilities played differing roles in academic performance across hospitality modules. In the majorly practical courses of *Food & Beverages workshops I* and *II, Wine & Spirits*, and *Rooms division*, the ability to manage other people’s emotions, measured by adaptive emotional conflict management strategies, contributed significantly to the final grades. The learning outcomes in these courses are adapted from industry-based skills and management competencies. They focus on skilling the students for hospitality-related management positions and developing their knowledge of field-related expertise (such as principles of sparkling wine or the main steps of winemaking). Moreover, the evaluation structure included group work. Deploying emotion management and regulation competencies are instrumental for teamwork collaboration. As MacCann et al. (2020) and Wilson-Wünsch et al. (2016) suggested, competent students successfully influence the emotions of others, which helps them build dependable bonds with classmates and develop supportive relationships with teachers (*Food & Beverages workshops I* and *II, Wine & Spirits*). Emotion management may also give the students an advantage in excelling in guest-oriented behavior (*Rooms division*). We should note that the mean score on the emotion management subtest (*M* = 0.45) was similar or lower compared to three samples of undergraduate psychology students in Schlegel and Mortillaro (2019). Considering the specific subject of the students who participated in this study and the importance of EI for a successful career in hospitality, it seems essential for students and young professionals to invest further in training this competence.

In addition to emotion management, understanding emotions was relevant in the module *Rooms division*. This module focused on teaching front-office skills and housekeeping. The courses rely on role-play as a learning method and contain lessons on conducting check-ins, talking to guests, and providing operational services. For such tasks and in the context of role plays, comprehending the causes and consequences of emotions is essential as it defines the success of the service delivery (Koc and Boz, 2019). Since front-office management and housekeeping involve following organizational guidelines, behavioral scripts, knowledge about how to conduct daily business, and the need for versatile execution, it was not surprising that fluid ability and conscientiousness emerged as additional predictors. A higher cognitive intelligence facilitates the acquisition of knowledge (Blair, 2006), and conscientious students may be more committed to completing tasks adequately and employ deeper, more strategic approaches to learning (Duff et al., 2004; Poropat, 2009; Wilson-Wünsch et al., 2016; Verbree et al., 2021). A learning-by-doing experience may enhance this relationship. Emotion management, complemented by emotion understanding, fluid ability, and conscientiousness, appears to make for a good set of emotional and technical competencies needed to provide services to guests. Our results might indicate that such learning settings (i.e., role-plays, group work, learning by practice) favor the leverage of emotional intelligence in learning new skills and knowledge.

While emotion management deals with the emotions of others, the ability to regulate emotions depicts how individuals influence their own emotions. This ability was relevant for the grade in *Wine & Spirits*, but in a somehow surprising direction: We found that a lower score on emotion regulation predicts a better grade. Alcoholic beverages can be used as a non-adaptive coping strategy, and indeed, the use of alcohol has been related to difficulties in emotion regulation (e.g., Dvorak et al., 2014). Though speculative, this may indicate that students with lower emotion regulation ability may be more open to engaging with the subject matter, which ultimately helps them obtain better grades in this module.

The modules *Food & Beverage workshops I* and *II* focus in detail on cuisine and kitchen operations, using didactic workshop formats that team up students in gastronomic environments. The evaluation structure includes theory-based dimensions assessing the knowledge related to the practice and foundations of the jobs (e.g., cooking methods, kitchen management principles, etc.) but also behavior and attitude components (e.g., punctuality, contribution to the team, etc.). Again, emotion management predicted better grades, likely reflecting the students’ capacity to manage peers in fast-paced and stressful teamwork situations. The learning purpose of these workshops is for students to experience being part of a kitchen brigade and participate in meal preparations and services at sales points. Therefore, the primary skills that the courses aim at developing are those of completing a task in a coordinated fashion for a specific deliverable under often stressful service delivery settings. Interestingly, a lower openness seemed important in *Food & Beverages workshops I*: When preparing dishes and creating new plates, too much creativity may be a pitfall, whereas sticking to the conventions may result in better grades.

Summed up, emotional abilities and personality were predictive of the final grades in modules presenting a professional environment concretely (involving medium to high stake interactions with peers, teachers, and guests). Yet another factor emerged: Age predicted better grades in *Food & Beverages workshops I*, *II,* and *Rooms division*. We can speculate that students who are a bit more mature tend to be more confident (Pearce, 2017) and use more efficient learning strategies (Wilson-Wünsch et al., 2016) that may be particularly beneficial in the more practical courses among these modules (age approached significance in *Wine & Spirits*). We further presume that age may also convey the accumulation of experience not as a student in hospitality but as a guest: In their twenties, young adults gain meaningful experiences traveling and dining out. These might lead them to use their private experiences to develop their professional skills.

### Theoretical modules

With a decrease in practical course activities, we observed a decline in the importance of emotional abilities. Instead, the theoretical aspects usually taught in more in-class settings, such as lectures and classroom exercises, emphasized a more substantial role of cognitive ability. Fluid ability includes the capacity to reason, infer relationships between objects, and is related to acquiring knowledge. This ability seems essential to grasp the abstract nature of quantitative data in business administration (*Introduction to business tools* which focuses on math and computing knowledge) and acquire behavioral scripts and guidelines (*Rooms division*). It seems important to note that in the module that involves interaction with guests (*Rooms division*), fluid ability and emotion management predicted the grades in unison. In contrast, in *Introduction to business tools*, fluid ability took the spotlight to explain this self-study module, followed by a lower openness that may help stay focused on going over the abstract material of the course and succeed in theory-focused exams (Verbree et al., 2021).

We could identify significant predictors for five of six modules. No significant predictors could be found for the *Introduction to hospitality management* grade. One reason might be that none of the variables considered were systematically relevant to performance in the courses in this module. However, this seems in contrast with our general findings. Instead, we suspect the reasons are statistical: The module grade had low reliability, which makes inferential analyses less trustworthy. The fact that this module includes language courses alongside topics in hospitality likely added noise to the data. In fact, removing the course *Introduction to business English* would have improved the reliability within this module to 0.613. An approach to disentangle potential effects could be to separately analyze course grades that differ substantially in the topic (e.g., management in restauration vs. management in the front office), in content (procedural knowledge vs. soft skills), and in didactic methodology (frontal lectures vs. interactive classes) in future studies.

### Limitations and future directions

Our study faces several limitations. An important one addresses low reliabilities on some module grades and psychometric measures. One reason may be heterogeneity across course grades, especially in *Introduction to hospitality management*. As discussed above, this may have been caused by varied contents conglomerated into one grade and the broad range of didactic methods. Additionally, some modules include teacher ratings of students’ transferable skills (e.g., teamwork), which may have somewhat inflated the associations we observed with emotional abilities. Future studies should ideally keep a more detailed record of each course’s methods and grading procedures. In particular, avenues for future research could explore the effect of assessment and didactic methods on the relationship between emotional competencies and academic performance. Such findings could be valuable for both the advancement of knowledge and practice, especially in courses that may demand more effort (tapping into conscientiousness), involve more difficult subject material (tapping into cognitive abilities), or rely heavily on human interaction (tapping into emotional abilities). Exploring the effect of the grading procedure and difficulty on the results could strengthen our understanding of the role of EI in learning and support higher education leadership in developing curricula that improve the learner’s readiness for professional challenges. Higher education is being challenged for its relevance in preparing its graduates. Therefore, designing innovative curricula that address social and professional needs, including training students’ EI, is crucial to developing a unique value proposition for higher education institutes.

Part of our sample consisted of international students whose first language was neither English nor French. The fact that some reliabilities are lower than those reported in other publications (Gosling et al., 2003; Schlegel and Mortillaro, 2019) might be ascribed to language barriers. Yet this could also be the case because the GECo measures emotional competencies using general workplace situations not explicitly related to the hospitality context, thus presenting the students with potentially unfamiliar work scenarios. Here, researchers may find slightly different results if they use a measure for emotional competencies with situations adapted to the context of hospitality. Unfortunately, to our knowledge, such a measure has yet to be made available.

Our findings may be dependent on the curriculum of the EHL. Further research should investigate whether the results hold across other institutions that educate in hospitality management. In this line, it may be interesting also to assess learning styles (Duff et al., 2004; Wilson-Wünsch et al., 2016), as well as relationship qualities with peers and teachers to shed more light onto the mechanisms through which emotional abilities affect the students’ grades (MacCann et al., 2020). Our results seem promising to elucidate that emotional competencies contribute primarily *via* the interactive parts of the curriculum, and fluid ability primarily *via* tasks that involve processing and reasoning. Other aspects of cognitive intelligence, such as crystallized abilities as an estimate of students’ wealth of semantic, acquired knowledge, can also be interesting to investigate to add further detail to this picture.

The students’ motivation, goals and stress management may impact their attitudes toward learning and thus moderate our findings (Behnke, 2012). Similarly, controlling for tendencies to avoid social interactions on the job seems important (Koc, 2019). Finally, we must also consider that school is a different environment than an actual hospitality business. Wilson-Wünsch et al. (2016) point out that a great deal of hospitality workers’ expertise (especially their cognitive performance) is honed in the field only after they leave school. How hospitality students’ cognitive and emotional abilities benefit not just grades but will also continue shape in the later workplace can be investigated with longitudinal studies. Such studies would add a theoretical grounding to the professional training methods and tools available to develop the workforce. Given the meta-analytic results provided by Miao and colleagues on how EI affects service quality (Miao et al., 2019) and job performance in general (Miao et al., 2021), we expect that the relationships are promising and deserve further attention.

## Conclusion

Our study demonstrated that emotional abilities, measured *via* a performance-based test instead of self-report, predicted hospitality grades beyond fluid ability and personality under the condition that courses involve practical work. These results provide valuable evidence that EI, when measured as an ability, explains unique and essential aspects of how students engage with a higher education environment that is replete with human interaction. Hospitality students draw from their emotional and cognitive (fluid) abilities to master the complex and diverse education that prepares them for careers in hotels, restaurants, and service industries. On the one hand, their abilities to manage, understand, and regulate emotions play important roles in interactive courses. On the other hand, modules that emphasize more abstract or theoretical material benefit from fluid abilities. Finally, for specific modules, students may have an easier time staying focused on following instructions, learning conventions, and developing skills if they are more conscientious and focused on current practices than on generating novel ideas.

The complexity of job demands in hospitality calls for an equally complex set of skills not limited to purely cognitive abilities but interpersonal competencies as well (Dominique-Ferreira et al., 2022). Our evidence suggests that while some aspects of personality seem important, researchers and educators should focus more on the underlying abilities necessary for acquiring knowledge and work experiences. Past studies have shown that emotional abilities can be trained (Hodzic et al., 2018), and hospitality professionals continue to express interest in fostering their staff’s emotional competencies (Wolfe and Kim, 2013; Wolfe et al., 2014). We thus reaffirm previous’ authors’ statements that hospitality education should implement EI courses and address emotional aspects in their coursework, to help students attain better professional achievement and social life in educational settings (Scott-Halsell et al., 2008; Wolfe, 2017).

## Data availability statement

The raw data supporting the conclusions of this article will be made available by the authors, without undue reservation.

## Ethics statement

Ethical review and approval was not required for the study on human participants in accordance with the local legislation and institutional requirements. The patients/participants provided their written informed consent to participate in this study.

## Author contributions

JV: data curation and writing – original draft. IB: resources, supervision, project administration, and writing – review and editing. MM: conceptualization, methodology, project administration, and writing – review and editing. All authors contributed to the article and approved the submitted version.

## Funding

This work was supported by Open access funding by University of Geneva.

## Conflict of interest

The authors declare that the research was conducted in the absence of any commercial or financial relationships that could be construed as a potential conflict of interest.

## Publisher’s note

All claims expressed in this article are solely those of the authors and do not necessarily represent those of their affiliated organizations, or those of the publisher, the editors and the reviewers. Any product that may be evaluated in this article, or claim that may be made by its manufacturer, is not guaranteed or endorsed by the publisher.

## Footnotes

^1^ https://www.qualtrics.com
